# Isolation of adult mouse microglia using their in vitro adherent properties

**DOI:** 10.1016/j.xpro.2021.100518

**Published:** 2021-05-06

**Authors:** Zoe Woolf, Taylor J. Stevenson, Kevin Lee, Yewon Jung, Thomas I.H. Park, Maurice A. Curtis, Johanna M. Montgomery, Michael Dragunow

**Affiliations:** 1 Department of Pharmacology, Faculty of Medical and Health Sciences, The University of Auckland, Auckland, Private Bag 92019, New Zealand; 2 Centre for Brain Research, The University of Auckland, Auckland, Private Bag 92019, New Zealand; 3 Department of Anatomy and Medical Imaging, The University of Auckland, Auckland, Private Bag 92019, New Zealand; 4 Department of Physiology, Faculty of Medical and Health Sciences, The University of Auckland, Auckland, Private Bag 92019, New Zealand

**Keywords:** Cell culture, Cell isolation, Neuroscience

## Abstract

Microglia are the primary innate immune effectors of the central nervous system. Although numerous protocols have been developed to isolate fetal mouse microglia, the isolation of adult mouse microglia has proven more difficult. Here, we present a simple, widely accessible protocol to isolate pure microglia cultures from 4- to 14-month-old mouse brains using their adherent properties *in vitro*. These isolated microglia recapitulate the adherent properties of adult human microglia and present a more suitable model for studying age-related diseases.

For complete details on the use and execution of this protocol in adult human microglia, please refer to Rustenhoven et al. (2016).

## Highlights

• An optimized protocol for the isolation of pure adult mouse microglia
• Utilization of the adherent properties of adult mouse microglia for isolation
• Isolated microglial cultures suitable for a range of functional assays

## Graphical abstract

**Figure:**
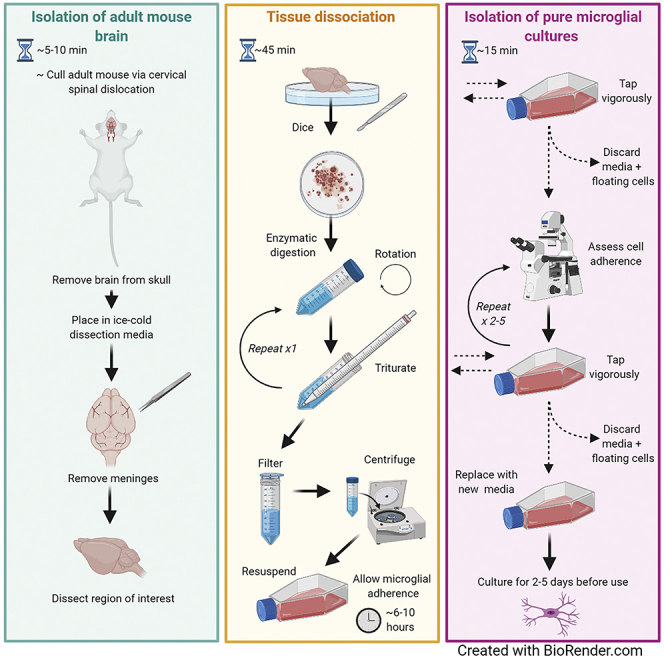

## Before you begin

The protocol below describes the specific steps for isolating pure microglia cultures from aged adult mouse brains (12–14 months). However, our laboratory has utilized this protocol in the isolation of microglial cultures from mice as young as 4 months. Our laboratory also uses the same protocol to isolate microglia from adult human brain biopsy samples (Rustenhoven et al., 2016).

**CRITICAL:** This protocol cannot be used for the isolation of microglia from newborn mouse pups (0–7 days old) due to the different adherent properties of microglia at this age.

### Preparation of dissection media

**Timing: [5 min]**

To 200 mL MEM (Gibco, CA, USA) add:

1. 1% (2 mL) Penicillin-Streptomycin solution (PS; Gibco, CA, USA)
2. 2.5% (5 mL) HEPES buffer solution (Gibco, CA, USA)
3. 1% (2 mL) 1M Tris stock solution ∗

Store at 4°C for up to one month.

∗Make 1M Tris buffer by dissolving Tris Base (Merck, MO, USA) in distilled water, adjust pH to 7.2 using 1M HCl.

***Note:*** Sterile-filter the combined solution using a 0.22 μm filter. Cool to 4°C before use.

### Preparation of complete media

**Timing: [5 min]**

To 500 mL DMEM/F12 (Gibco, CA, USA) add:

4. 10% (55 mL) fetal bovine serum (FBS; Moregate, Australia)
5. 1% (5.5 mL) penicillin-streptomycin (PS; Gibco, CA, USA)
6. 1% (5.5 mL) GlutaMAX ® (Gibco, CA, USA)

Store at 4°C for up to one month. Warm for approximately 20 min in 37°C water bath prior to use.

### Preparation of enzyme digestion mix

**Timing: [5 min]**

To prepare 10 mL of enzyme digestion mix per gram of tissue (see table below)

7. Add 5 mL EBSS (at 20°C–25°C) to one vial of lyophilized papain and gently mix to dissolve
8. Prepare a DNase stock at a concentration of 1000 U/ mL by diluting DNase I (20,000 U/ mL, Invitrogen) in HBSS. Stock can be stored at −20°C for up to 12 months.
9. To 8.65 mL of hibernate A medium, add 1.25 mL dissolved papain, and 100 uL of DNase stock
10. Warm to 37°C prior to use

***Note:*** Do not store, use within 1 h of preparation.

**Table:** Enzyme digestion mix

Reagent	Final Concentration	Amount
Lyophilized Papain dissolved in 5 mL EBSS (20 U/mL)	2.5 U/ mL	1.25 mL
DNase (1000 U/ mL)	10 U/ mL	100 μL
Hibernate A Medium	n/a	8.65 mL
**Total**	**n/a**	**10 mL**

## Key resources table

**Table:** 

REAGENT or RESOURCE	SOURCE	IDENTIFIER
**Antibodies**		

Anti-Iba1 (1:1000)	Abcam	Cat#Ab5076, RRID: AB_2224402
Anti-PU.1 (1:500)	Cell Signaling	Cat#2258, RRID: AB_2186909
Anti-GFAP (1:30,000)	Abcam	Cat# Ab4674, RRID:AB_304558
PE-P2RY12	BioLegend	Cat#392103, RRID:AB_2716006

**Critical commercial assays**		

Click-iT ™Edu Cell Proliferation Kit	Invitrogen	C10340

**Experimental models: organisms/strains**		

Mouse strain: C57/BL6J (4–14 months, M/F)	The Jackson Laboratory	N/A

**Other**		

EBSS	Invitrogen	14155-063
HBSS	Invitrogen	14025-092
DNase I	Invitrogen	18047-019
Papain	Worthington Biochemical Corporation	LK003176
Hibernate-A medium	Invitrogen	A12475-01
DMEM/F12 medium	Gibco	11320033
MEM, GlutaMAX™ Supplement	Gibco	42360-032
HEPES (1M)	Gibco	15630080
Tris Base	Merck	10708976001
PS	Gibco	15140
FBS	Moregate	0820
GlutaMax ® (100×)	Invitrogen	35050-038
0.5% Trypsin-EDTA	Gibco	15400054

EBSS (Earle’s Balanced Salt Solution), HBSS (Hank's Balanced Salt Solution), DMEM (Dulbecco’s Modified Eagle’s Medium), MEM (Minimum Essential Media), HEPES (N-2-hydroxyethylpiperazine-N-2-ethane sulfonic acid), PS (Penicillin-Streptomycin), FBS (Fetal Bovine Serum), EDTA (Ethylenediaminetetraacetic acid).

## Step-by-step method details

### Isolation of adult mouse brain

**Timing: [5–10 min]**

Immediately isolate the brain of a freshly culled adult C57/BL6J mouse, and dissect the brain region of interest from which you wish to culture microglia.

1. Quickly euthanize adult mouse via cervical dislocation

**CRITICAL:** All animal handling and euthanasia must be conducted in accordance with guidelines approved by an animal ethics committee.

***Note:*** The following steps (2–4) should be carried out as quickly as possible (ideally <5 min), to minimize cell death.

2. Decapitate the mouse, and immediately immerse the head in 80% ethanol for sterilization
3. Remove the skin and skull using sterile dissection tools (Troubleshooting 1)
4. Carefully remove the brain and place it into a petri dish containing ice-cold (4°C) dissection media
5. Remove the meninges

***Note:*** Failure to remove meninges may result in contamination of cultures with unwanted cell types. Remove the meninges quickly using sharp forceps under the dissecting microscope. Other studies (Roqué and Costa, 2017) have achieved this via rolling the tissue over sterile filter paper, albeit in neonatal brain tissues.

6. Dissect brain region of interest

***Note:*** Here we only collected forebrain tissues for culturing, removing, and discarding the mid- and hind-brain, as well as the hippocampus.

### Dissociation of brain tissue

**Timing: [45 min]**

Dissociate the tissue via mechanical and enzymatic dissociation, filter, and re-suspend tissue-containing media into a flask.

***Note:*** These steps must be carried out inside a biological safety hood.

7. Place tissue into a pre-weighed falcon tube containing ice cold HBSS and record weight of tissue
8. Transfer tissue to a sterile petri dish and wash with ice cold HBSS
9. Dice the tissue into small cubes < 1 mm^3^ using a sterile scalpel
10. Using 5 mL at a time, add enzyme digestion mixture to the diced tissue in the petri-dish, collecting the media containing tissue fragments, and transferring this to a sterile Falcon tube. Use 10 mL of enzyme digestion mix per gram of tissue.

**CRITICAL:** For ≤1 *g* of tissue, use a 10 mL Falcon tube, for ≥ 1 *g* of tissue, use a 50 mL Falcon tube. This is to ensure the tissue remains submerged in the enzyme digestion mixture during step 11.

11. Incubate for 15 min at 37°C whilst applying gentle agitation

***Note:*** We routinely use a MACSmix™ Tube Rotator, although equivalents can be used.

12. Triturate the mixture ∼10 times, and re-incubate for a further 15 min at 37°C with gentle rotation
13. Triturate the mixture a further ∼10 times

**CRITICAL:** Use a 10 mL strip pipette, and avoid being too vigorous with triturations as this can cause a decrease in cell viability (Troubleshooting 2).

14. Allow undigested tissue to settle to the bottom, and then collect 5 mL of tissue-containing media at a time, passing this through a sterile 70 μm Nylon mesh filter into another sterile 50 mL Falcon tube
15. Pass a further 10 mL of warm complete media (per 1g of tissue) through the filter to rinse, making a final volume of 20 mL per 1 *g* of tissue
16. Centrifuge the cell suspension at 170 × *g* for 5 min
17. Remove the supernatant by gentle pipetting
18. Re-suspend in 10 or 15 mL of complete media, and transfer into a non-coated T25 or T75 tissue culture flask, respectively. Incubate cells at 37°C, 5% CO_2_

***Note:*** Depending on the weight of the brain, or if you are pooling brains in a single culture preparation, choose either a T25 or T75 flask to re-suspend the cells. For the isolation of cells from a single brain, we recommend using a T25 flask. For the isolation of cells from pooled brains (>2), we recommend using a T75 flask.

### Isolation of pure microglial cultures

**Timing: [15 min]**

∗6–10 h later∗

Isolate microglia by repeated tapping to remove non-adherent cells, leaving a pure culture of adherent, adult mouse microglia.

19. Check the T25 or T75 flask for microglia adherence using a bright field microscope. Microglia will appear phase bright and adherent to the bottom of the flask

**Figure 1:**
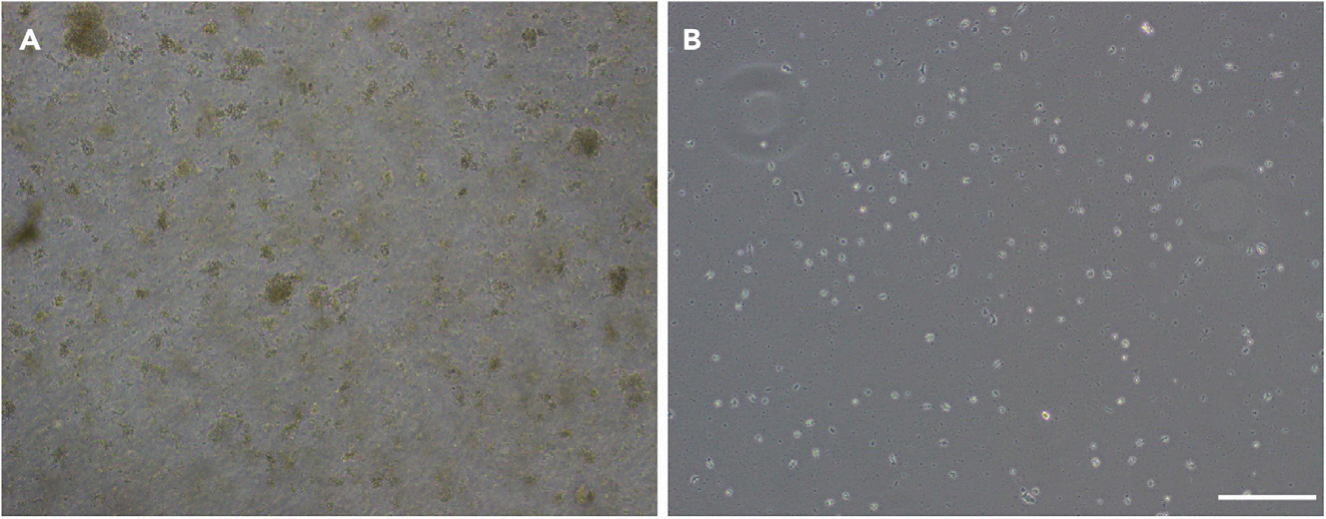
Debris removal by media changing Representative images of cell culture before (A) and after (B) full media change. Images taken on Olympus CKX53 bright field inverted microscope, 10× objective. Scale bar, 200 μm.

***Note:*** Microglia may not be visible in some cultures due to a large amount of debris. In these cases, perform 1–2 full media changes of the flask with warmed complete media before observing microglial adherence (Figure 1).

20. Tap the flask firmly and repeatedly to dissociate non-microglial cells

**CRITICAL:** Be careful not to allow any fluid to get into the filter at the neck of the flask.

**Figure 2:**
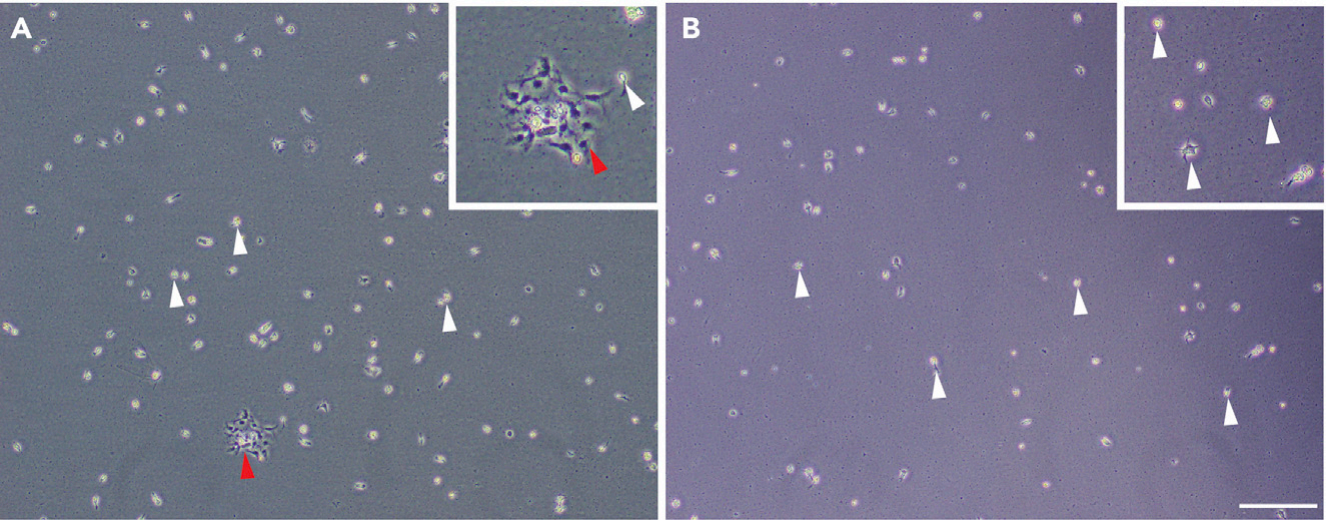
Tapping to remove non-adherent cells and achieve pure microglial cultures Representative phase-images of cultures prior to tapping (A) containing non phase-bright pericytes (red arrows) and phase-bright microglia (white arrows). Following tapping (B), only phase bright microglia are present (white arrows). Images taken on Olympus CKX53 bright field inverted microscope, 10× objective. Scale bar, 200 μm.

21. Check for any adherent cells which are not microglia (non-phase bright cells) under the microscope (Figure 2A)

22. Remove all media containing floating non-adherent cells, discard, and replace with fresh, warmed complete media
23. Repeat step 20–22 until only phase bright microglia appear, with all cells adherent to the bottom of the flask (Figure 2B)

***Note:*** Depending on the weight of tissue dissociated, this may take 2–5 washes to achieve a pure culture.

**CRITICAL:** The purity of the microglial cultures will be highly dependent on how rigorously this step is performed. Ensuring that all non-adherent cells are tapped and washed off prevents contamination of cultures with other cells such as pericytes, but slightly decreases overall yield. Therefore, this step can be adjusted based on priority of yield vs purity.

24. Incubate pure microglia cultures for 2–5 days in fresh complete media at 37°C, 5% CO2

***Note:*** Microglial cultures in T25 or T75 flasks should not need media changing in this time-frame, but if media appears discolored, replace ½ of the media with fresh complete media.

### Harvesting and plating

**Timing: [30 min]**

**Figure 3:**
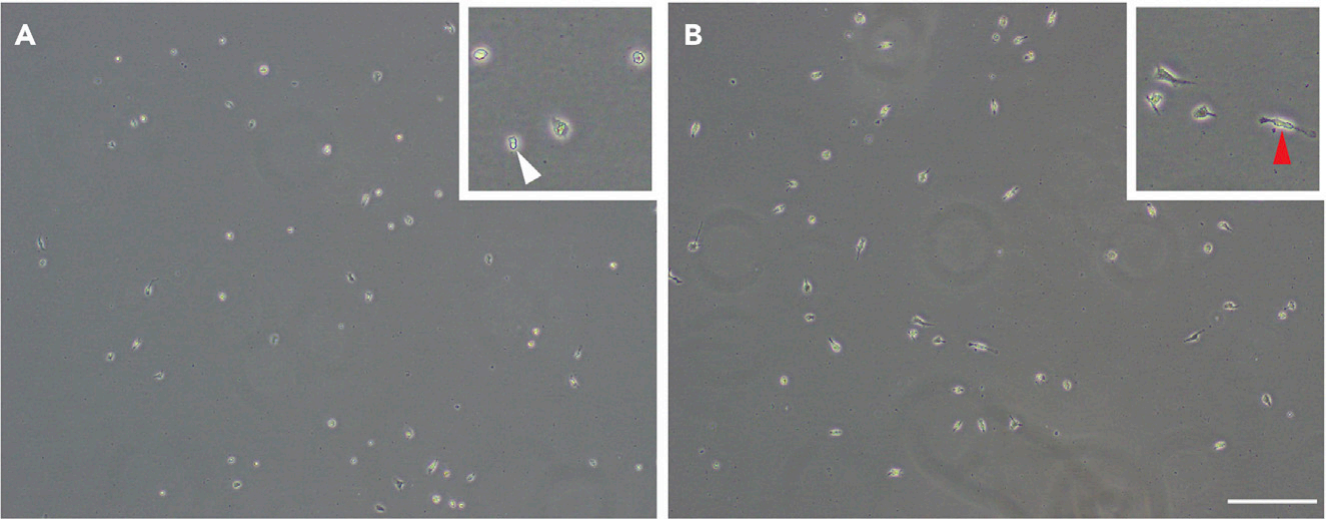
Microglia must reach rod-like and ramified morphologies before harvesting Representative images of microglia which are still rounded (white arrow) and not yet ready for harvesting (A), and cells which have spread out into rod-like and ramified morphologies (red arrow) (B) and may be harvested for experimental use. Images taken on Olympus CKX53 bright field inverted microscope, 10× objective. Scale bar, 200 μm.

25. After 2 days, observe microglia under the microscope to assess morphology prior to harvesting for plating. Cells can be harvested if the microglia have spread out into rod-like and ramified morphologies (Figure 3B)

**CRITICAL:** Harvesting microglia too early (Figure 3A) may result in low yield or cell death during plating or use in assays (Troubleshooting 2).

26. Remove all media from T25 or T75 flask
27. Add 1 mL (T25) or 3 mL (T75) of 0.5% Trypsin-EDTA (warmed to 37°C) and incubate for 2 min at 37°C
28. Repeatedly firmly tap flask to aid in microglial detachment

***Note:*** Observe microglial detachment under the microscope, and if microglia are still adherent to the flask, proceed to step 29. If all microglia appear detached, proceed to step 30.

**CRITICAL:** It is advised to use 0.5% Trypsin-EDTA at this step, rather than the more commonly used 0.05%–0.25% Trypsin-EDTA. This is due to the highly adherent nature of adult mouse microglia in culture. Use of stronger trypsin often completely negates the need for step 29 (cell scraping), which has adverse effects on cell yield.

29. Using a sterile rubber cell scraper, gently pass the scraper over the bottom of the flask. Check for microglial detachment under the microscope. If microglia are still adherent, pass the scraper over an additional time to detach these cells
30. Add 4mL (T25) or 7 mL (T75) of complete media to stop the Trypsin-EDTA reaction
31. Collect the 5 or 10 mL media containing the microglial cell suspension, and transfer to a sterile falcon tube
32. Centrifuge at 170 × *g* for 5 min
33. Remove the supernatant and re-suspend the cell pellet. Cells can be counted at this point, and subsequently used for plating, flow cytometry, or other experimental assays

***Note:*** Isolated adult mouse microglia can be plated in 96, 48, 24 or 6 well plates with no poly-D-lysine (PDL) or matrigel coating required due to their adherent properties but appear healthier when plated in larger wells. We recommend plating in 48 well plates for functional assays, at a density between 1.6 × 10^4^ and 3.2 × 10^4^ cells/cm^2^.

**Figure 4:**
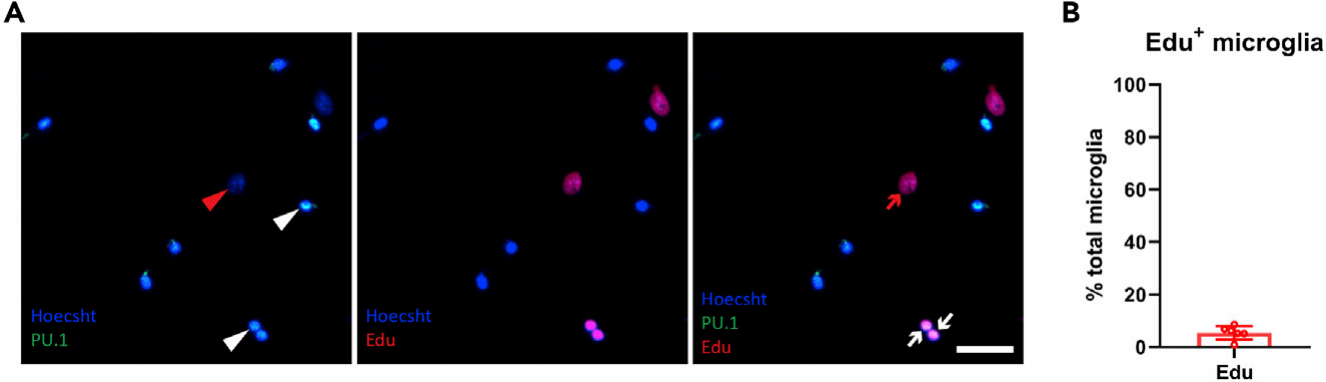
Isolated adult mouse microglia have a low proliferative capacity *in vitro* Representative fluorescent images of microglial cultures following the addition of Edu for 48 h (A). White arrowheads represent PU.1 positive microglia, whilst white arrows represent PU.1^+^ Edu^+^, dividing microglia. Red arrowheads represent PU.1^-^ cells, whilst red arrows represent PU.1^-^ Edu^+^ dividing non-microglial cells, which are largely pericytes. Scale bar, 50 μm. Average percentage of Edu+ microglia (PU.1^+^) per case, n = 6 adult mouse brain isolations (B). All data are plotted as mean ± SEM.

***Note:*** Like adult human microglia, adult mouse microglia have a limited proliferative capacity *in vitro* with approximately 5.5% Edu^+^ microglia present over 48 h (Figure 4). Therefore, these cells cannot be passaged or re-plated, but can remain in culture for 1–2 weeks depending on the purity of the original culture.

## Expected outcomes

**Figure 5:**
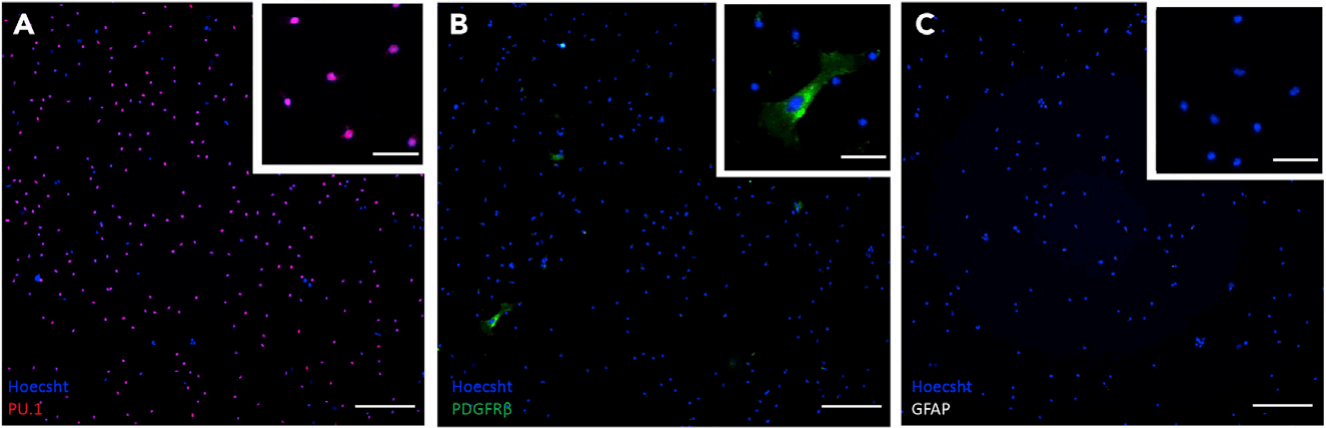
Isolated mouse microglial cultures are PU.1 positive, PDGFRβ and GFAP negative Representative fluorescent images showing mouse microglial cultures are positive for the nuclear microglial marker PU.1 (A), show minimal labeling for the pericyte marker PDGFRβ (B), and are negative for the astrocytic marker GFAP (C), indicating high purity cultures. Scale bar, 200 μm, 50 μm zoom.

**Figure 6:**
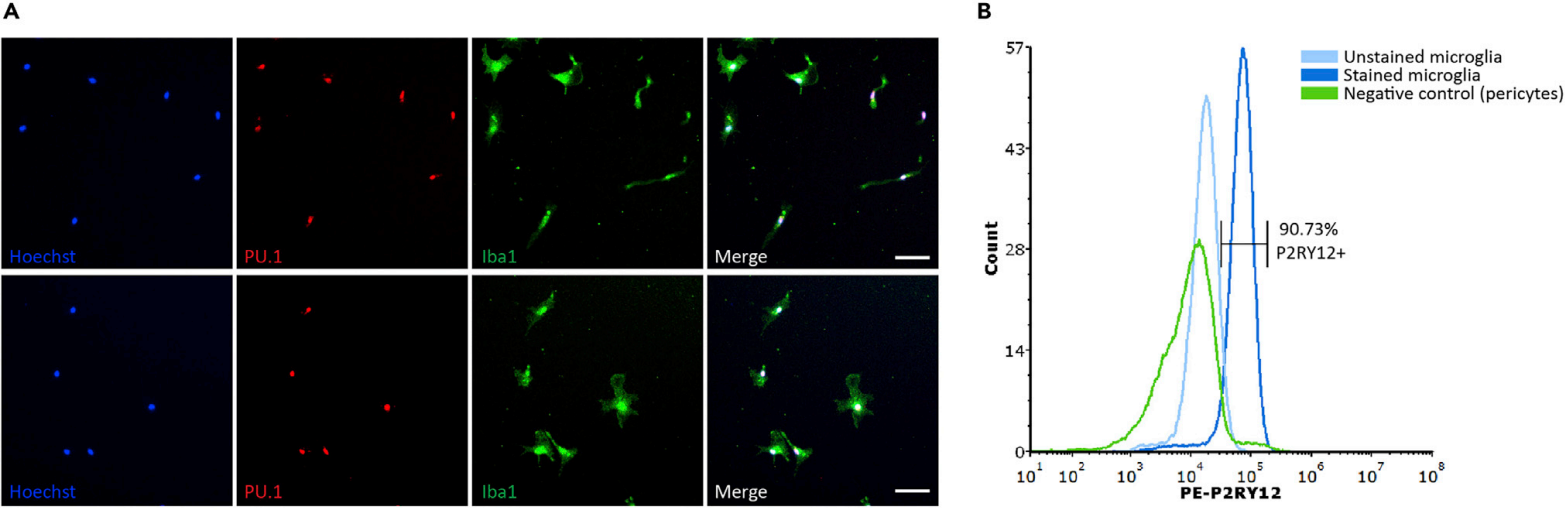
Isolated mouse microglia express macrophage markers PU.1 and Iba1, and the microglial-specific marker P2RY12 Representative fluorescent images showing mouse microglial cultures are positive for the nuclear microglial marker PU.1, and cytoplasmic protein Iba1 (A). Microglia adopt morphologies ranging from rod-like to ramified. Scale bar, 50 μm. P2RY12 expression by isolated mouse microglia and pericytes via flow cytometry showing microglial purity (B).

**Figure 7:**
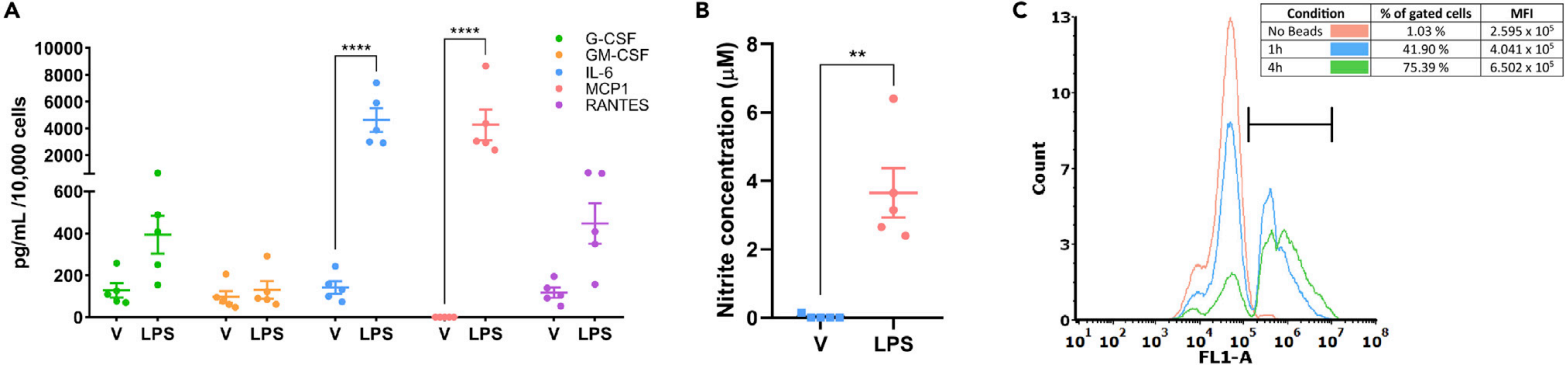
Isolated mouse microglia are suitable for a range of functional assays Isolated mouse microglia were treated with either vehicle (0.1% BSA in PBS) or 10 ng/mL of LPS for 24 h and conditioned media collected. Concentrations of cytokines (G-CSF, GM-CSF, IL-6, MCP1 and RANTES) were determined by cytometric bead array (A). 2-way ANOVA, p∗∗∗∗ < 0.0001. Concentrations of NO were determined by Griess assay (B). Mann-Whitney test, ∗∗p= 0.0079. All data are plotted as mean ± SEM. n = 5 individual mouse brains. Isolated microglia were incubated with FluoSpheres™ Polystyrene Microspheres (1 μm) beads for 4h or 1h. Cells were washed thoroughly to remove uninternalized beads and collected by trypsinization. Phagocytosis of beads was determined by a rightward shift in FL1 intensity via flow cytometry (C).

Here, we demonstrate a simple, reproducible, and widely accessible protocol for the isolation of adult mouse microglia, with no requirement for special equipment such as that needed for magnetic activated cell sorting (Bohlen et al., 2017, Bordt et al., 2020). Using this isolation protocol, high purity (>80% PU.1^+^) microglial cultures can be readily obtained from adult mouse brains aged 4–14 months (Figure 5). Isolated microglia express pan-macrophage markers PU.1 and Iba1, in addition to the microglial-specific marker P2RY12 (Figure 6), and adopt rod-like and ramified morphologies in culture (Figure 5). These cells exist in basally resting state, with no secretion of nitric oxide (NO), and minimal secretion of inflammatory cytokines and chemokines (Figures 7A and 7B). Importantly, these cells can be induced with LPS treatment, showing enhanced secretion of inflammatory mediators (Figures 7A and 7B). These isolated microglia also actively phagocytose fluorescent beads, and therefore present as a suitable model for a range of *in vitro* functional studies (Figure 7C).

**Figure 8:**
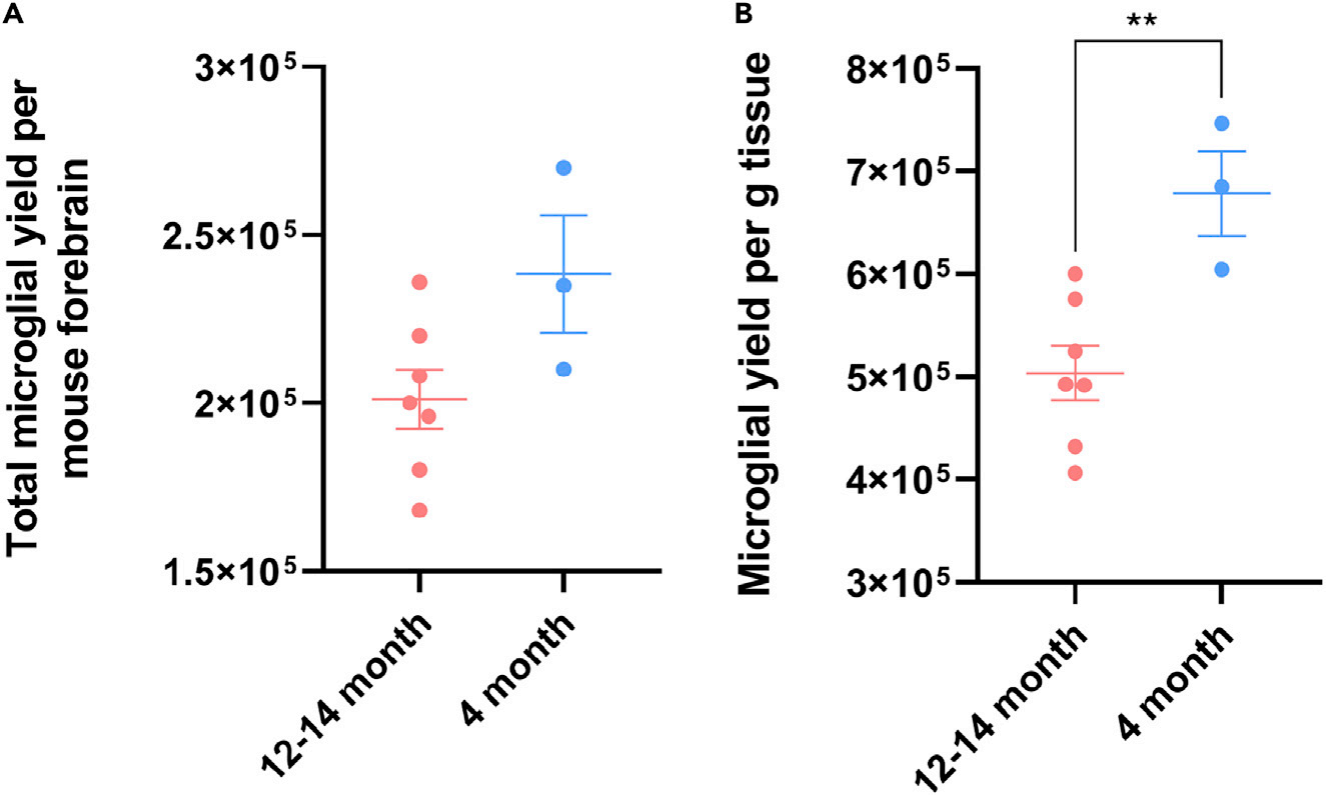
Average microglial yield from adult mouse brain varies with age Expected average microglial yield from 12–14, and 4 month old mouse forebrains. Total microglial yield per mouse forebrain in 12–14 and 4 month old mice (A). Total microglial yield, normalized to tissue weight in 12–14 and 4 month old mouse forebrains (n = 7, n = 3) (B). ∗∗ = p < 0.0045. Student’s T test. All data are plotted as mean ± SEM.

The expected microglial yield ranges from approximately 170,000 to 270,000 per adult mouse forebrain. Isolation from younger mouse brains (4 months) yields approximately 680,000 cells per gram of brain tissue, whilst aged mouse brains (12–14 months) yields approximately 503,000 cells per gram of brain tissue (Figure 8). Importantly, this protocol allows the isolation of microglia from aged mice, which if used between 10–24 months is considered comparable with middle-older aged humans (38–69 years) (Jackson et al., 2017). As such, this isolation protocol provides a useful model for studying aged mouse microglia.

## Quantification and statistical analysis

Total microglial yields per mouse forebrain (Figure 8A) were grouped (12–14 month and 4 month) and compared using an unpaired Students T test (p = 0.0634). Microglial yields were then normalized to give the yield per gram of tissue (total microglial yield per forebrain / forebrain weight × 1000) (Figure 8B). The 12–14 month and 4 month groups were then compared using an unpaired Students T test (p = 0.0071).

CBA secretome data were normalized to pg/ml / 10,000 cells, and was analyzed using a 2-way ANOVA (Sidak’s multiple comparison test), comparing vehicle to LPS (Figure 7A). Nitrite concentration, determined by Griess assay, was analyzed using a Mann-Whitney test (Figure 7B).

## Limitations

### Yield

Although the microglial yields achieved using this protocol are sufficient for a range of studies, the yield does not permit high-throughput assays. This can be attributed to both the low proliferative capacity of adult microglia in culture (Figure 4), and the age of the mice used. Using this protocol, we found the microglial yield from aged mice (12–14 months) to be significantly lower than that from 4 month old mice (Figure 8). Therefore, for experiments requiring large microglial numbers, we recommend using younger adult mice (2–4 months), or for aged microglial studies, pooling multiple mouse brains.

### Method of culling

Although not possible in our laboratory due to the nature of receiving the animals, where possible it is best to euthanize mice via isofluorine and clear blood vessels using an ice-cold saline solution to prevent possible contamination of cultures with peripherally circulating macrophages. However, the isolated microglial cultures presented utilizing our protocol express P2RY12 (Figure 6B), a microglial specific marker, indicating minimal contamination with peripheral macrophages.

### Purity with long-term culturing

Although high purity microglial cultures can be obtained using this protocol, the maintenance of cultures for an extended period of time (> 7 days) can lead to a decline in culture purity. This deterioration in purity is directly dependent on the number of pericytes present in the original culture. As pericytes are proliferative cells *in vitro*, over time, these cells can divide to contaminate the culture. Therefore, caution must be taken in long-term experimental use of these cultures. Culture purity can be assessed throughout the culturing using a phase-microscope to observe cell size and morphology, with microglia existing as phase-bright rod-shaped or ramified cells, and pericytes appearing as phase-dim, large, flat cells (Figures 2 and 3).

### Use of serum-containing media

Our laboratory routinely uses media containing 10% FBS for the culturing of both mouse and human derived microglia. Recent studies have demonstrated that serum-cultured microglia display altered gene expression profiles and are basally activated *in vitro* (Bohlen et al., 2017). However, following our protocol, isolated mouse microglia are seen to exist in a basally resting state, showing minimal secretion of inflammatory cytokines, and no secretion of NO (Figure 7). Despite our data showing negligible activation, the presence of FBS within culture media may be considered a potential limitation; therefore, at the discretion of the reader, microglia may alternatively be cultured in TIC media as previously detailed (Bohlen et al., 2017).

## Troubleshooting

### Problem 1

Contamination by fur, blood or external contaminants during dissection may result in contaminated isolated cultures.

### Potential solution

Spray 70%–80% ethanol repeatedly onto the head both prior to, and following, decapitation. Ensure the use of separate tools and gloves after handling the animal’s fur and blood (steps 2–6).

### Problem 2

Significant microglial cell death during isolation or harvesting.

### Potential solution

If you observe significant cell death, ensure the initial steps of mouse culling and brain isolation are carried out rapidly (steps 2–4). Additionally, try using less vigorous trituration steps (steps 12–13). Before harvesting, it is important to ensure the microglia are spread out into rod-like and ramified morphologies (step 25). In cases where there was a larger volume of tissue, or where the initial culture was particularly high in debris, microglia may take an additional 2–3 days to adopt a morphologically ‘resting’ appearance (Figure 3B). Harvesting these cells too early can result in cell death and a loss in cell number. Moreover, it is important to allow the microglia to reach a resting state before harvesting to ensure these cells are not pre-activated for use in inflammatory studies.

### Problem 3

Low purity cultures (<80% microglia) following isolation.

### Potential solution

Steps 19–23 are critical in obtaining high purity microglial cultures. Unlike fetal mouse microglia, adult mouse microglia are highly adherent *in vitro*, and readily adhere to the bottom of plastic flasks. Therefore, it is essential to ensure firm tapping to dislodge all non-adherent cells (Figure 2A). If cultures appear to have lower purity and pericyte contamination despite these steps, we recommend leaving microglia to adhere for a shorter amount of time (6 h). Longer periods between steps 18 and 19 allows a greater time period for less-adherent cells, such as pericytes, to adhere, and therefore decreases culture purity.

### Problem 4

The cell strainer may become blocked if the brain tissue is not properly digested.

### Potential solution

To troubleshoot this problem, ensure the tissue is well diced (step 9), and ensure trituration steps are carried out thoroughly. Triturations can be increased up to 15 times (steps 12 and 13) if required. Agitation/rotation of the tissue (step 11 and 12) can also be increased to aid in digestion.

### Problem 5

Lack of microglial adherence to the bottom of the flask during isolation may result in a low yield.

### Potential solution

Lack of microglial adherence to the flask following tissue digestion may occur if the tissue suspension is particularly heavy with debris (appears very cloudy), resulting in debris preventing cells from adhering to the base of the flask. This often occurs if a large amount of tissue is seeded into a flask that is too small (step 18). In such cases, it is possible to re-seed the media containing the tissue digestion into a new flask after the first harvest to increase total yield. To achieve this, instead of discarding the media at step 22, re-seed this media into a fresh T25 or T75 flask and allow a further 6 h for cells to adhere before continuing on from step 19 as previously described.

## Resource availability

### Lead contact

Further information and requests for resources and reagents should be directed to and will be fulfilled by the lead contact, Michael Dragunow (m.dragunow@auckland.ac.nz).

### Materials availability

This study did not generate new unique reagents.

### Data and code availability

The published article includes all datasets generated or analyzed during this study.
